# Gait Stability Characteristics in Able-Bodied Individuals During Self-paced Inclined Treadmill Walking: Within-Subject Repeated-Measures Study

**DOI:** 10.2196/42769

**Published:** 2023-06-05

**Authors:** Chenmiao Lu, Rawan Al-Juaid, Mohammad Al-Amri

**Affiliations:** 1 Department of Rehabilitation Medicine The First Affiliated Hospital of Zhejiang University School of Medicine Zhejiang China; 2 Physical Therapy Department Almuwaih General Hospital Ministry of Health Taif Saudi Arabia; 3 School of Healthcare Science Cardiff University Cardiff United Kingdom

**Keywords:** healthy individuals, muscle activation, self-paced walking, slope walking, stability, treadmill-based gait analysis, virtual reality

## Abstract

**Background:**

Inclined walking is a challenging task that requires active neuromuscular control to maintain stability. However, the adaptive strategies that preserve stability during inclined walking are not well understood. Investigating the effects of self-paced inclined treadmill walking on gait stability characteristics and the activation patterns of key lower limb muscles can provide insights into these strategies.

**Objective:**

The aim of this study was to investigate the effects of self-paced inclined treadmill walking on gait stability characteristics and the activation of key lower limb muscles.

**Methods:**

Twenty-eight able-bodied individuals (mean age 25.02, SD 2.06 years) walked on an augmented instrumented treadmill for 3 minutes at 3 inclination angles (−8°, 0°, and 8°) at their preferred walking speed. Changes in gait characteristics (ie, stability, walking speed, spatial-temporal, kinematic, and muscle forces) across inclination angles were assessed using a repeated measures ANOVA and the Friedman test.

**Results:**

The study revealed that inclined treadmill walking has a significant impact on gait characteristics (*P*<.001). Changes were observed in spatial-temporal parameters, joint angles, and muscle activations depending on the treadmill inclination. Specifically, stability and walking speed decreased significantly during uphill walking, indicating that it was the most challenging walking condition. Uphill walking also led to a decrease in spatial parameters by at least 13.53% and a 5.26% to 10.96% increase in temporal parameters. Furthermore, joint kinematics and peak activation of several muscles, including the hamstrings (biceps femoris, long head=109.5%, biceps femoris, short head=53.3%, semimembranosus=98.9%, semitendinosus=90.9%), gastrocnemius (medial gastrocnemius=40.6%, lateral gastrocnemius=35.3%), and vastii muscles (vastus intermedius=12.8%, vastus lateralis=16.7%) increased significantly during uphill walking. In contrast, downhill walking resulted in bilateral reductions in spatial-temporal gait parameters, with knee flexion increasing and hip flexion and ankle dorsiflexion decreasing. The peak activation of antagonist muscles, such as the quadriceps, tibialis anterior, and tibialis posterior, significantly increased during downhill walking (rectus femoris=97.7%, vastus lateralis =70.6%, vastus intermedius=68.7%, tibialis anterior=72%, tibialis posterior=107.1%).

**Conclusions:**

Our findings demonstrate that able-bodied individuals adopt specific walking patterns during inclined treadmill walking to maintain a comfortable and safe walking performance. The results suggest that inclined treadmill walking has the potential to serve as a functional assessment and rehabilitation tool for gait stability by targeting muscle training. Future research should investigate the effects of inclined treadmill walking on individuals with gait impairments and the potential benefits of targeted muscle training. A better understanding of the adaptive strategies used during inclined walking may lead to the development of more effective rehabilitation interventions for individuals with lower limb injuries.

## Introduction

Inclined walking is a challenging daily mobility activity that requires complex adaptation to maintain gait stability compared with level walking [1-3]. Evidence suggests that inclined walking decreases gait stability even in healthy individuals [4,5] and thus poses a higher risk of falling than walking on stairs with a similar inclination [6]. Gait stability can be estimated by means of the gait stability ratio (GSR), which is estimated as the ratio of cadence to walking speed [5,7] and has been proven as a good indicator of stability and a potential predictor of falls [7,8]. For example, Ferraro et al [5] reported that more adaptations were required in an attempt to preserve gait stability during uphill walking compared to level walking in healthy elderly individuals (mean age 77.8, SD 4.8 years) by using the GSR. To date, gait stability characteristics in relation to kinematic parameters and muscular adaptations have not been established in healthy individuals. This warrants further observations to develop a comprehensive understanding of gait stability and associated adaptive strategies.

Other biomechanical literature has shown the impact of inclined walking on gait performance, including changes in postural adaptations [9], kinetics and kinematics parameters [10,11], joint work [3], and muscle activity [1,11-14]. Nevertheless, most studies have been limited by the difficulty of obtaining enough gait measurements due to a small number of continuous strides on instrumented ramps [3,10,14,15] or on fixed-speed treadmills [1,9,12,13]. Furthermore, muscle activity on inclined surfaces has been commonly measured by using electromyography (EMG) [1,11-14]; despite its reliability, EMG has some limitations that cannot be ignored. First, the accuracy of the acquired data can be greatly affected by the displacement of the EMG electrodes during inclined walking [16]. Second, the decision to use EMG requires selecting only a finite number of large superficial muscles from both sides [17], which does not provide an overall idea about muscular adaptation on inclined walking surfaces. Taken together, there is a need to examine gait stability alongside more meaningful gait parameters including the activity of key muscles during comfortable walking speed.

Fortunately, technological advancements in instrumented treadmills can be advantageous in overcoming many limitations in previous literature. These cutting-edge treadmills are laboratory based, designed with split belts, and equipped with 2 embedded force plates [18]. They differ from conventional treadmills as they automatically adjust the belt speed in real time to match the participant’s self-paced walking [19,20]. Meanwhile, they allow for the collection of data on hundreds of continuous strides during walking within a virtual reality (VR) environment [18,19,21]. Recent evidence supports that self-paced walking within a VR environment would facilitate a natural walking experience, which in turn enables the collection of valid gait parameters that resemble overground walking [18-21]. To improve the use of these instrumented treadmills, a human body model (HBM) has been developed and validated to allow the estimation of kinetic and kinematic parameters in real time [22]. The HBM also estimates the forces of several muscles, which can be a promising alternative approach to EMG for understanding the role of key muscles in walking performance. Despite the growing use of instrumented treadmills, limited studies have been conducted using inclined gait analysis during self-paced treadmill walking [19,20,23], and only 1 study has employed the HBM model, specifically on children with cerebral palsy [23]. The self-paced treadmill study by Kimel-Naor et al [19] proposed that speed–inclination interactions can provide a comprehensive picture of slope walking. However, to our knowledge, no published study has looked at gait stability and associated muscular adaptations during self-paced treadmill walking at different inclinations.

Therefore, this study aimed to investigate the effects of inclination angle during self-paced treadmill walking on gait characteristics (ie, gait stability, walking speed, lower limb joint kinematics, and spatiotemporal parameters) and on the peak activations of key lower limb muscles. In this study, we sought to use the novelty of the gait real-time analysis interactive lab (GRAIL) system to collect rich, highly accurate, and reliable gait data in real time during a self-paced inclined treadmill walking within a VR environment. We estimated gait stability by calculating the GSR, and we employed the HBM model to obtain a more comprehensive understanding of the walking patterns and muscular adaptations in healthy individuals during self-paced inclined treadmill walking. This study would yield valuable knowledge on gait stability and adaptive strategies during walking at different inclinations.

## Methods

### Study Design

A within-subject repeated-measures study design was used.

### Participants

Twenty-eight able-bodied adults (age 25.02, SD 2.06 years; 14 men and 14 women) participated voluntarily in this study, which took place at the Cardiff University School of Healthcare Sciences. Participants were recruited from Cardiff University and local communities. Interested individuals who did not have any medical, musculoskeletal, neurological, cardiovascular, or vestibular impairments that may affect their gait were invited to sign a written informed consent prior to participation. The protocol of this study was approved by the Cardiff University School of Healthcare Sciences Research Ethics Committee.

### Setting

3D kinetics, joint kinematics, and spatiotemporal parameters were collected bilaterally using the GRAIL (Motek Medical BV) system at Cardiff University. The GRAIL system comprises an instrumented dual-belt treadmill with 2 embedded force platforms, a VR system projected onto a 180° screen, and a 10-camera motion tracking system (Vicon; Oxford Metrics). The speed of the 2 belts was set to the self-paced mode for each participant, as described by Sloot et al [24]. Additionally, the speed of the visual flow synchronized with the treadmill speed. For safety considerations, a nonbodyweight support harness was worn by the participant throughout the walking trials. The harness was suspended from the ceiling, and its position did not interfere with the participant’s natural walking movement.

### Experimental Procedure

All participants attended a single session at our movement analysis laboratory. Twenty-five reflective markers were placed on the participant’s body according to the HBM (Motek) lower limb marker set [22]. The calibration process began by asking the participant to stand still in a T-pose on the treadmill in order to create a skeleton file in the computer. When the operator gave the order “start,” the participant began walking while looking straight ahead at the screen. After calibration, each participant was familiarized with the self-paced walking mode by asking them to accelerate and decelerate their walking speed according to the operator’s orders. Following the operator’s instructions, each participant had at least 3 minutes to practice the walking experiments before recording the outcomes. The participant was then given a minute to warm up and establish a comfortable walking speed. Depending on the walking condition, the slope angle would then gradually change to either up- or downslope by automatic computer control. Downhill walking, level walking, and uphill walking conditions were simulated when the platform inclination was adjusted to −8°, 0°, and 8°, respectively. Once the slope angle reached 8° or −8°, the participant sustained walking for 2 minutes. After the slope walking conditions, the inclination angle of the treadmill returned to the horizontal level for 30 seconds to return to the baseline state.

Each participant completed a 3-minute self-paced walking trial under each of the 3 walking conditions in random order. The 3 trials were performed consecutively with at least 2 minutes of rest in between to allow for recovery. Participants were not given any instructions during walking trials, and talking was not permitted in the laboratory to avoid any distractions that might affect their walking performance.

### Data Processing

This study used the HBM model that is embedded in D-Flow software (version 3.00, Motek Medical BV) to estimate all gait parameters, including muscle force, stride length, and joint angles. Estimated muscle activation levels (*F*/*F*_max_) were calculated based on moment arms, joint moments, skeletal pose, and polynomial equations for 11 muscles around the knee joint bilaterally [22]. Various gait parameters were processed during the 3 walking conditions, including GSR, walking speed, max lower limb joint kinematics, and spatiotemporal parameters. Gait data were processed for all participants, but 1 participant’s data was eliminated due to missing information. We primarily focused on the muscles around the knee joint, including the quadriceps, hamstrings, gastrocnemius, soleus, and tibialis muscles, as we hypothesized that these muscles would activate differently during level, uphill, and downhill walking. The peak muscle activation levels of these muscles were further processed in Matrix Laboratories (MATLAB version R2017, The MathWorks Inc).

### Statistical Analysis

The analysis aimed to compare gait characteristics and muscle activation levels across the 3 walking conditions. Therefore, a repeated-measures ANOVA and the Friedman test were used to explore the effect of inclines on lower limb muscle activity and gait parameters after testing the normality of the data by the Shapiro-Wilk test. The level of statistical significance was set at *P*<.05. If significant effects were obtained, post hoc analyses were performed to contrast between the 3 conditions and locate the significant changes. SPSS (version 25.0 for Macintosh; IBM Corp) was used to perform all statistical analysis.

### Ethics Approval

This study involved human participants; informed consent was obtained from all participants in the study and all procedures were approved by the Cardiff University School of Healthcare Sciences Research Ethics Committee. Informed consent was obtained from all participants, and the original consent allows for secondary analysis of the research data without additional consent. Participants’ privacy and confidentiality were strictly protected by anonymizing and coding the collected data to prevent identification. No compensation was offered for participation in the study, as participants were recruited on a voluntary basis.

## Results

### Spatial-Temporal Parameters

During uphill walking, all spatial-temporal gait parameters changed significantly compared to level walking. Inverse relationships were noted between parameters, such as a significant increase in GSR by 15.58% during uphill walking and a significant reduction in walking speed by 23.14%. Additionally, spatial parameters decreased significantly by at least 13.53% during uphill walking compared to level walking, while temporal parameters significantly increased by 5.26%-10.96%.

During downhill walking, almost all spatial-temporal gait parameters showed bilateral reductions compared to level walking, except for GSR values, which increased by 3.46%. The mean walking speed during downhill walking did not show a significant change relative to the mean speed during level walking (Table 1).

**Table 1:** Mean spatial-temporal gait parameters on both sides across walking conditions.

Parameter	Side	DW,^a^ mean (SD)	LW,^b^ mean (SD)	UW,^c^ mean (SD)	*P* value	Mean percentage difference (%)^d^		
						1-2	2-3	1-3
GSR^e^ (steps/m)		1.47 (0.13)	1.42 (0.12)	1.66 (0.20)	<.001	3.46	15.58^f^	12.14^f^
Walking speed (m/s)		1.34 (0.16)	1.35 (0.20)	1.07 (0.19)	<.001	0.74	23.14^f^	22.41^f^
Stride length (m)^g^	R	1.37 (0.12)	1.42 (0.12)	1.23 (0.15)	<.001	3.58	14.34^f^	10.77^f^
	L	1.37 (0.12)	1.42 (0.12)	1.23 (0.15)		3.58	14.34^f^	10.77^f^
Step length (m)^g^	R	0.69 (0.06)	0.71 (0.06)	0.61 (0.08)	<.001	2.86	15.15^f^	12.31^f^
	L	0.68 (0.06)	0.71 (0.06)	0.62 (0.08)		4.32	13.53^f^	9.23^f^
Stride time (s)^g^	R	1.03 (0.08)	1.06 (0.09)	1.16 (0.12)	<.001	4.32	9.01^f^	11.87^f^
	L	1.03 (0.08)	1.06 (0.09)	1.16 (0.12)		4.32	9.01^f^	11.87^f^
Stance time (s)^g^	R	0.65 (0.05)	0.69 (0.06)	0.77 (0.10)	<.001	5.97^f^	10.96^f^	16.9^f^
	L	0.66 (0.05)	0.69 (0.06)	0.77 (0.09)		4.44^f^	10.96^f^	15.38^f^
Swing time (s)^g^	R	0.38 (0.03)	0.37 (0.02)	0.39 (0.03)	.002	2.67	5.26^f^	2.6^f^
	L	0.37 (0.03)	0.37 (0.03)	0.39 (0.03)		0	5.26^f^	5.26^f^

^a^DW: downhill walking.

^b^LW: level walking.

^c^UW: uphill walking.

^d^Mean percentage difference (%): 1=downhill, 2=level, 3=uphill walking.

^e^GSR: gait stability ratio.

^f^Significant post hoc at *P*<.05.

^g^No significant differences between right and left sides.

### Joint Kinematics

**Figure 1:**
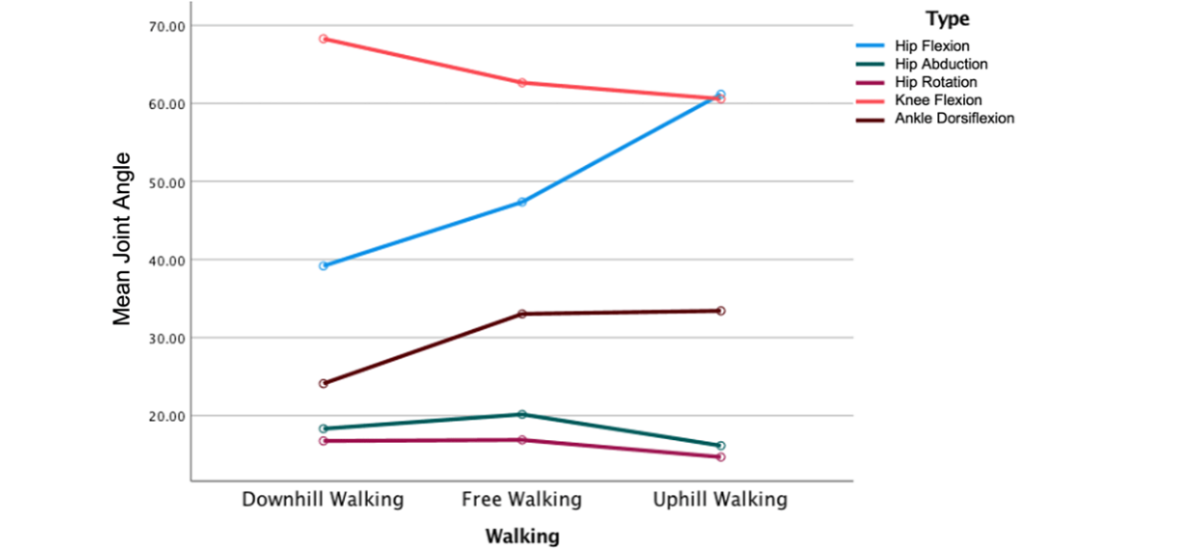
Mean joint angles across walking conditions.

During uphill walking, the mean max hip flexion significantly increased by over 25% on both sides compared to level walking (*P*<.001). However, mean max hip abduction, hip rotation, and knee flexion were significantly reduced during uphill walking compared to level walking (*P*<.001, *P*=.02, and *P*=.04, respectively). During downhill walking, the mean max knee flexion significantly increased from about 62° during level walking to about 68° during downhill walking (*P*<.001), while mean hip flexion and ankle dorsiflexion were significantly reduced (*P*<.001). The results did not show significant differences in joint angle kinematics between both sides across walking conditions (Figure 1).

### Muscle Activations

During uphill walking, most muscles showed a significant increase in peak activation, including the hamstrings (ie, the long head [LH] and short head [SH] of the biceps femoris [BF], semimembranosus, and semitendinosus), quadriceps, and gastrocnemius (all *P*<.001, except vastus intermedius *P*=.03, and vastus lateralis *P*=.02). Interestingly, however, the rectus femoris (RF) was the only quadriceps muscle that showed significantly less activation bilaterally during uphill walking compared to level walking (*P*<.001), while both tibialis anterior (TA) and tibialis posterior (TP) muscles showed a significant decrease in activation bilaterally during uphill walking (*P*<.001).

During downhill walking, the quadriceps, TA, and TP muscles showed a significant increase in mean activation in both lower limbs (*P*<.001), with RF showing the highest peak activation among all muscles. The hamstrings, gastrocnemius, and soleus muscles were significantly less activated during downhill walking compared to level walking. Notably, the peak activation of TP was significantly higher (*P*<.001) on the right side than the left side (mean peak activation during downhill walking was 0.86, SD 0.31 *F*/*F*_max_ on the right side and was 0.47, SD 0.34 *F*/*F*_max_ on the left side).

## Discussion

### Principal Findings

This study established the impacts of self-paced inclined treadmill walking on gait stability characteristics and muscle activation levels during downhill, level, and uphill walking conditions. Comprehensive gait data were collected bilaterally during self-paced treadmill walking within a naturalistic VR environment (detailed in Multimedia Appendix 1), which offers additional insights into the adaptive neuromuscular strategies that maintain stability during walking on inclined surfaces.

Our findings suggest that walking on inclined surfaces requires gait adaptation to enhance stability, as evidenced by the significant changes in all spatiotemporal parameters, maximium lower limb joint kinematics, and the peak activation of all muscles across walking conditions. In both inclined walking conditions, participants showed less stability, as demonstrated by higher GSR means compared to level walking (Table 1). Of all walking conditions, gait stability and walking speed were significantly reduced during uphill walking. Participants took significantly shorter and slower strides with significantly more time spent in the stance phase during uphill walking compared to other walking conditions, suggesting that uphill walking was the most challenging condition for our participants. Reduced walking speed is often used as a stability-enhancing strategy [20]. In a similar study, Kimel-Naor et al [19] reported that participants (mean age 31.8, SD 4.3 years) walked slower and exhibited more kinematical changes in lower extremity joint angles during self-paced uphill than downhill treadmill walking [19]. However, they did not assess gait stability or muscle activity on inclined treadmill walking [19]. In line with this study, Ferraro et al [5] reported that healthy elderly adults had a significant increase in their GSR and a significant decrease in their step length, cadence, and walking speed during walking on a 10° inclined overground ramp [5]. Our findings expand on this body of literature by establishing insightful biomechanical analysis of the impact of inclined treadmill walking on gait stability characteristics in relation to joint angles and muscular adaptations, and this is demonstrated as follows.

In this study, we observed two opposite biomechanical patterns associated with the direction of the incline when compared to level walking. During uphill walking, we found an average of 60° at both hip and knee joints and 34° dorsiflexion at the ankle joint were required in an attempt to bring the body’s center of mass forward. The greater hip and knee flexion elicited a significant concentric activation of the hamstrings (BF-LH=109.5%, BF-SH=53.3%, semimembranosus=98.9%, semitendinosus=90.9%) more than those of the quadriceps (vastus intermedius [VI]=12.8% and vastus lateralis [VL]=16.7%) and the plantar flexors (medial gastrocnemius=40.6%, lateral gastrocnemius=35.3%). These muscular adaptations would support the pronounced role of the hamstrings in absorbing power from the trunk and producing forward momentum in both legs to propel the body up the inclined surface [12]. During downhill walking, on the contrary, we found a significant reduction in flexion at both hip and ankle joints (mean max 39° and 24°, respectively), while knee flexion was significantly increased by 8.5%. These biomechanical adjustments are apparently needed during downhill walking because the center of mass usually moves in front of the sagittal plane [25], so it has to be pushed slightly back to counteract the effect of gravity. Consequently, these biomechanical adjustments elicited significant eccentric contraction of the quadriceps, tibialis anterior, and tibialis posterior during downhill walking compared to level walking (RF=97.7%, VL=70.6%, VI=68.7%, TA=72%, TP=107.1%). Our findings are generally in line with previous studies, which reported similar changes in gait parameters and muscular contributions during uphill and downhill walking conditions [9,12,13]. For example, Leroux et al [9] examined postural changes during treadmill walking at 5 inclinations (0%, ±5%, and ±10%) and they reported that uphill walking induced flexion posture in the hip, knee, and ankle joints and forward tilt of the pelvis and trunk, whereas downhill walking required less flexion posture at the 3 main lower extremity joints [9]. Pickle et al [12] and Franz and Kram [13] pointed out the role of the proximal muscles in power generation during uphill walking, and the role of the knee extensors in power absorption to preserve dynamic stability while lowering the body on a declined surface. Surprisingly, our study found that during downhill walking, the tibialis posterior muscle was significantly more activated on the right side (the dominant side of our participants), which may be due to the natural asymmetry of human gait and the dominant limb being used more frequently during certain tasks [18]. This asymmetry in muscle activation could reflect a compensatory mechanism for maintaining balance during downhill walking. Further research should delve deeper into this muscle to identify if the difference between the right and left side muscle activation always exists and the reason behind this phenomenon.

Our findings suggest that inclined treadmill walking can be a valuable tool for assessing gait stability and has potential to be incorporated into clinical assessments, particularly for patients at risk of falling during inclined walking, neuromuscular training, and fall prevention programs in rehabilitation. However, our results should be interpreted cautiously, since we only studied the effects of self-paced inclined treadmill walking in young, able-bodied participants and did not measure hip and ankle muscles, which limits the generalizability of our findings. To overcome this limitation, future research should recruit individuals with impaired gait stability, such as older adults or those recovering from an injury or illness, and measure other muscle groups. Additionally, determining the minimal clinically important difference in gait stability ratio or other parameters to prevent falls could be a promising avenue for future studies. Our study provides a baseline for future research investigating the effects of different types of inclined walking on gait stability in diverse populations.

### Conclusions

This study provides evidence that inclined treadmill walking is a challenging activity that requires adaptive neuromuscular control strategies to preserve stability. Results showed that the inclination angle of the treadmill significantly affected overall gait characteristics, reflecting changes in gait parameters and muscle activations. Of all walking conditions, uphill walking had the greatest impact on gait stability and walking speed reduction. Our findings suggest that inclined treadmill walking may serve as a functional assessment tool or rehabilitation intervention to improve gait stability and strengthen specific muscle groups. Further research is needed to investigate the effects of inclined treadmill walking on other patient populations and to identify the potential benefits of targeted muscle training.

## Appendix

Multimedia Appendix 1 Comprehensive gait data.

## Abbreviations

BF: biceps femoris
EMG: electromyography
GRAIL: gait real-time analysis interactive lab
GSR: gait stability ratio
HBM: human body model
LH: long head
RF: rectus femoris
SH: short head
TA: tibialis anterior
TP: tibialis posterior
VI: vastus intermedius
VL: vastus lateralis
VR: virtual reality

## Data Availability

The data sets generated and analyzed during this study are available from the corresponding author on reasonable request.
