# Supplementary material for: Gait Stability Characteristics in Able-Bodied Individuals During Self-paced Inclined Treadmill Walking: Within-Subject Repeated-Measures Study
Source: JMIR Form Res. 2023 Jun 5;7:e42769. doi: 10.2196/42769 (PMC10280335; doi:10.2196/42769)
Supplement: Multimedia Appendix 1 [file formative_v7i1e42769_app1.docx]

**Appendix of supplementary file**

Table 1: Bilateral joint angles across walking conditions.

| Parameter | Side | DW | LW | UW | *P* value | Mean Percentage difference (%)^b^ | | |
| --- | --- | --- | --- | --- | --- | --- | --- | --- |
|  |  | Mean ± SD | Mean ± SD | Mean ± SD |  | 1-2 | 2-3 | 1-3 |
| Hip Flexion^a^ | R | 39.25 ± 4.6 | 47.21 ± 3.5 | 61.15 ± 4.5 | <.001 | 18.4^c^ | 25.7^c^ | 43.6^c^ |
|  | L | 39.10 ± 5.9 | 47.52 ± 4.0 | 61.20 ± 5.5 |  | 19.4^c^ | 25.2^c^ | 44.1^c^ |
| Hip Abduction^a^ | R | 18.34 ± 3.3 | 20.26 ± 2.8 | 16.45 ± 2.9 | <.001 | 9.95 | 20.8^c^ | 10.9^c^ |
|  | L | 18.29 ± 3.6 | 20.05 ± 3.1 | 15.85 ± 2.9 |  | 9.2 | 23.4^c^ | 14.3^c^ |
| Hip Rotation^a^ | R | 16.72 ± 3.8 | 16.96 ± 5.1 | 14.04 ± 5.2 | .03 | 1.4 | 18.8^c^ | 17.4^c^ |
|  | L | 16.78 ± 4.2 | 16.79 ± 5.4 | 15.33 ± 5.0 |  | 0.1 | 9.1^c^ | 9.0^c^ |
| Knee Flexion^a^ | R | 68.16 ± 4.3 | 62.59 ± 3.2 | 60.98 ± 4.7 | <.001 | 8.5^c^ | 2.6^c^ | 11.1^c^ |
|  | L | 68.42 ± 3.1 | 62.72 ± 3.6 | 60.19 ± 6.5 |  | 8.7^c^ | 4.1^c^ | 12.8^c^ |
| Ankle Dorsiflexion^a^ | R | 24.70 ± 4.4 | 33.84 ± 5.0 | 34.31 ± 4.6 | <.001 | 31.2^c^ | 1.4 | 32.6^c^ |
|  | L | 23.49 ± 2.9 | 32.21 ± 4.1 | 32.53 ± 3.3 |  | 31.3^c^ | 0.1 | 32.3^c^ |

^a^No significant differences between right and left sides. ^b^Mean Percentage Difference: 1= Downhill, 2= Level, 3= Uphill walking.

^c^Significant post-hoc at *P*<.05.

Table 2: Bilateral peak muscle activation across walking conditions.

| Muscle | Side | DW | LW | UW | *P* value | Mean Percentage difference (%)^b^ | | |
| --- | --- | --- | --- | --- | --- | --- | --- | --- |
|  |  | Mean (SD) | Mean (SD) | Mean (SD) |  | 1-2 | 2-3 | 1-3 |
| Biceps Femoris LH | R | .13 ± .06 | .19 ± .06 | .65 ± .49 | <.001 | 37.5^c^ | 109.5^c^ | 133.3^c^ |
|  | L | .13 ± .07 | .17 ± .05 | .66 ± .46 |  | 26.7^c^ | 118.1^c^ | 134.2^c^ |
| Biceps Femoris SH | R | .08 ± .04 | .11 ± .04 | .19 ± .12 | <.001 | 31.6^c^ | 53.3^c^ | 81.5^c^ |
|  | L | .08 ± .04 | .11 ± .05 | .19 ± .11 |  | 31.6^c^ | 53.3^c^ | 81.5^c^ |
| Soleus | R | .28 ± .14 | .42 ± .11 | .40 ± .10 | <.001 | 40.0^c^ | 4.9 | 35.3^c^ |
|  | L | .23 ± .11 | .40 ± .10 | .43 ± .09 |  | 53.97^c^ | 7.2 | 60.6^c^ |
| Semimembranosus | R | .20 ± .09 | .24 ± .08 | .71 ± .68 | <.001 | 18.2^c^ | 98.95^c^ | 112.1^c^ |
|  | L | .19 ± .09 | .23 ± .08 | .78 ± .65 |  | 19.1^c^ | 108.9^c^ | 121.7^c^ |
| Semitendinosus | R | .10 ± .05 | .12 ± .04 | .32 ± .32 | <.001 | 18.2^c^ | 90.9^c^ | 104.8^c^ |
|  | L | .09 ± .05 | .11 ± .04 | .36 ± .31 |  | 20.0^c^ | 106.4^c^ | 120.0^c^ |
| Lateral Gastrocnemius | R | .18 ± .08 | .42 ± .13 | .60 ± .26 | <.001 | 80.0^c^ | 35.3^c^ | 107.7^c^ |
|  | L | .14 ± .07 | .45 ± .14 | .67 ± .25 |  | 105.1^c^ | 39.3^c^ | 130.9^c^ |
| Medial Gastrocnemius | R | .20 ± .09 | .53 ± .16 | .80 ± .30 | <.001 | 90.4^c^ | 40.6^c^ | 120.0^c^ |
|  | L | .16 ± .08 | .52 ± .16 | .77 ± .28 |  | 105.9^c^ | 38.8^c^ | 131.2^c^ |
| Tibialis Anterior | R | 1.02 ± .40 | .48 ± .17 | .18 ± .10 | <.001 | 72.0^c^ | 90.9^c^ | 140.0^c^ |
|  | L | .93 ± .38 | .39 ± .13 | .10 ± .06 |  | 81.8^c^ | 118.4^c^ | 161.2^c^ |
| Tibialis Posterior^a^ | R | .86 ± .31 | .26 ± .13 | .13 ± .05 | <.001 | 107.1^c^ | 66.7^c^ | 147.5^c^ |
|  | L | .47 ± .34 | .12 ± .07 | .08 ± .05 |  | 118.6^c^ | 40.0^c^ | 141.8^c^ |
| Rectus Femoris | R | 1.28 ± .84 | .44 ± .14 | .16 ± .08 | <.001 | 97.7^c^ | 93.3^c^ | 155.6^c^ |
|  | L | 1.43 ± .83 | .43 ± .15 | .09 ± .05 |  | 107.5^c^ | 130.8^c^ | 176.3^c^ |
| Vastus Intermedius | R | .45 ± .14 | .22 ± .10 | .25 ± .13 | <.001 | 68.7^c^ | 12.8^c^ | 57.1^c^ |
|  | L | .45 ± .13 | .22 ± .10 | .26 ± .13 |  | 68.7^c^ | 16.7^c^ | 53.5^c^ |
| Vastus Lateralis | R | .46 ± .15 | .22 ± .10 | .26 ± .14 | <.001 | 70.6^c^ | 16.7^c^ | 55.6^c^ |
|  | L | .43 ± .13 | .22 ± .09 | .25 ± .13 |  | 64.6^c^ | 12.8^c^ | 52.94^c^ |

^a^There was a significant differences between the activation levels of tibialis posterior in both sides (*P* <.001). ^b^Mean Percentage Difference: 1= Downhill, 2= Level, 3= Uphill walking. ^c^Significant post-hoc at *P*<.05.
